# Supplementary figures and images for: Combinations of genes at the 16p11.2 and 22q11.2 CNVs contribute to neurobehavioral traits
Source: PLoS Genet. 2023 Jun 2;19(6):e1010780. doi: 10.1371/journal.pgen.1010780 (PMC10266672; doi:10.1371/journal.pgen.1010780)

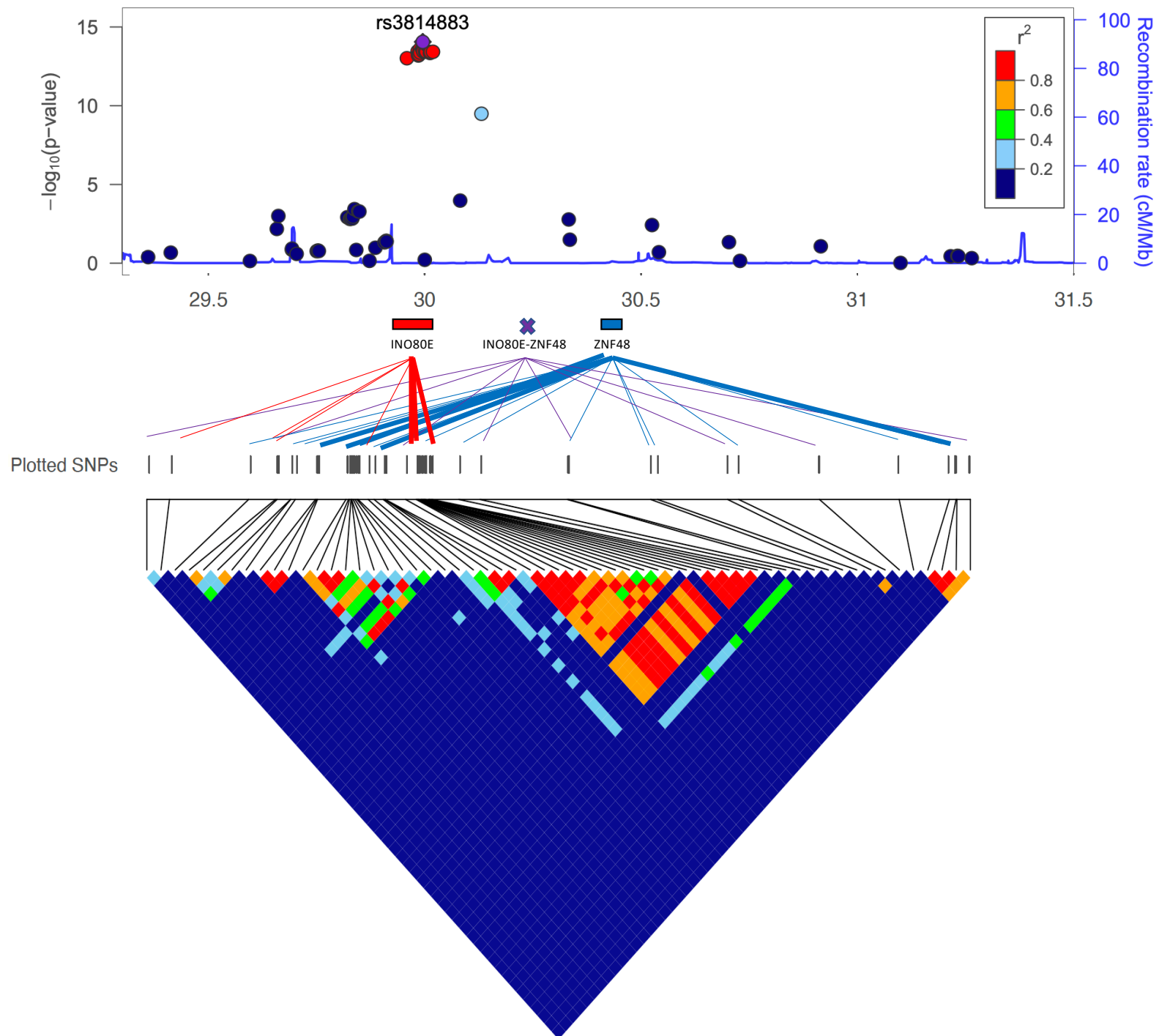

Supplement: S1 Fig — As a representative example of the relationship between the prediction SNP architecture for single vs. pairwise genes, we selected INO80E and ZNF48, the top schizophrenia pair, in the frontal cortex, a putative schizophrenia-relevant tissue. 60 SNPs from three categories are plotted: 20 INO80E predictors, 32 ZNF48 predictors, and 8 predictors of the INO80E/ZNF48 pair. Top: LocusZoom plot of association of predictor SNPs with schizophrenia. Circles: SNP predictors in one of the three (above) categories. Y-axis: schizophrenia association P-value (left, circles); recombination rate (right, blue line). X-axis: distance on chr. 16. Middle: Distribution of plotted SNP positions for all three categories (vertical bars) with colored lines representing prediction for gene or pair. Red rectangle: INO80E gene, red lines: INO80E predictors; blue rectangle: ZNF48 gene, blue lines: ZNF48 predictors; purple x: INO80E-ZNF48 theoretical expression pair, purple lines, INO80E-ZNF48 pair predictors. Bottom: LD structure of predictive SNPs. LD heatmap color scale is in the same order as the R2 scale in LocusZoom. Note: Approximately 120 additional unique genes (both coding and noncoding) are located in the region (not shown), including 30 between INO80E and ZNF48. (PDF) [file pgen.1010780.s001.pdf]

# ASD 16p

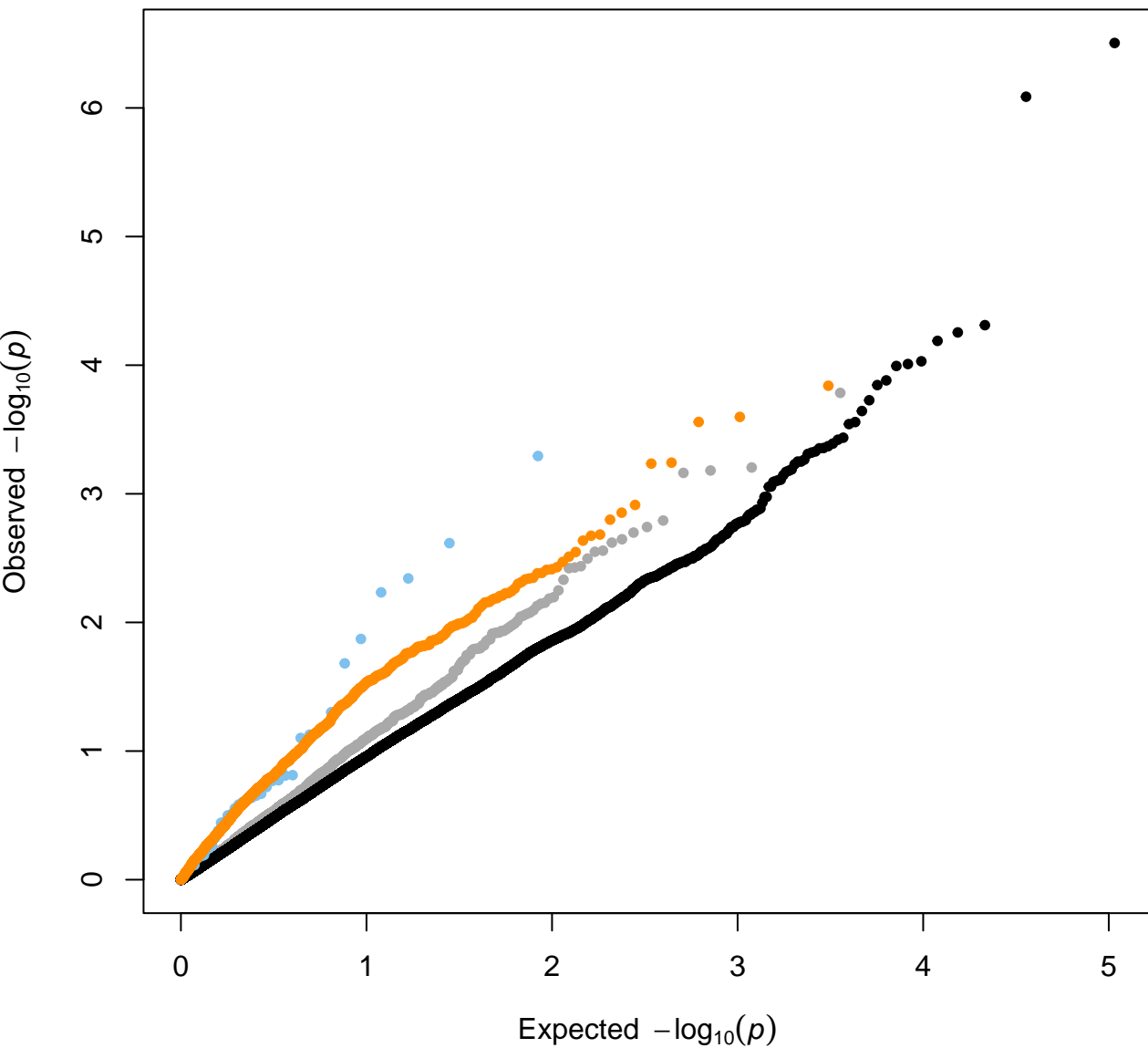

# BIP 16p

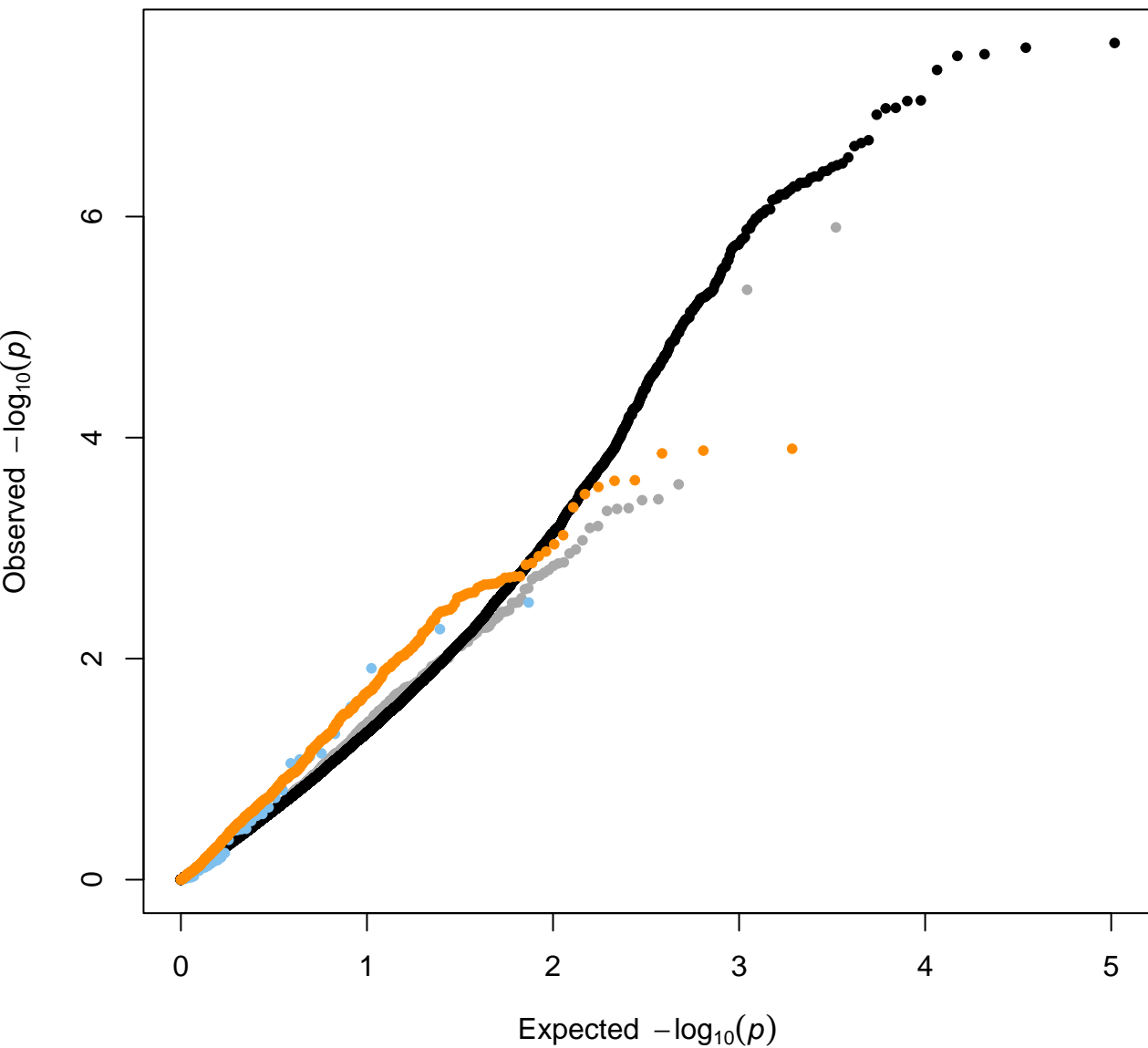

# SCZ 16p

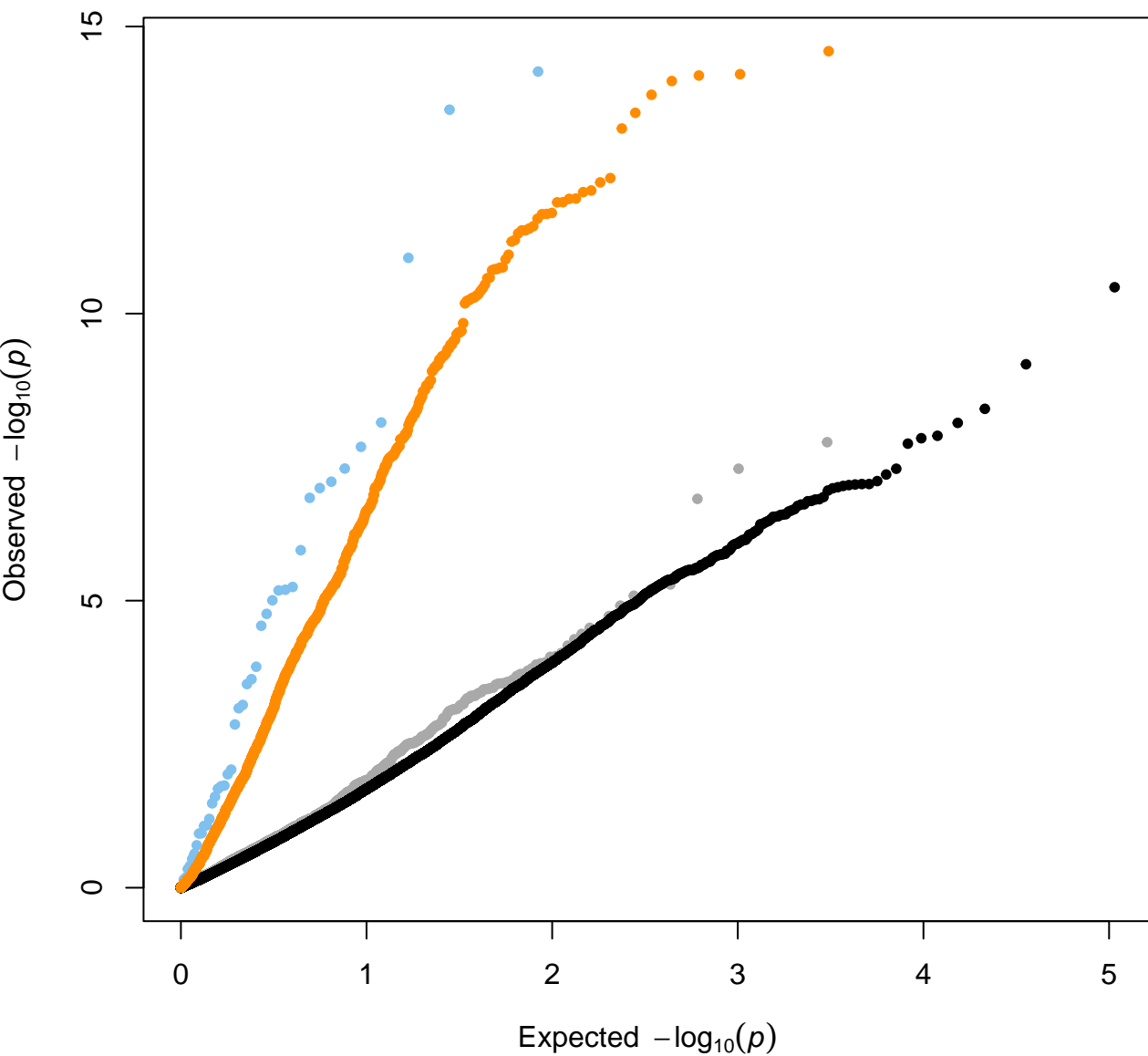

# BMI 16p

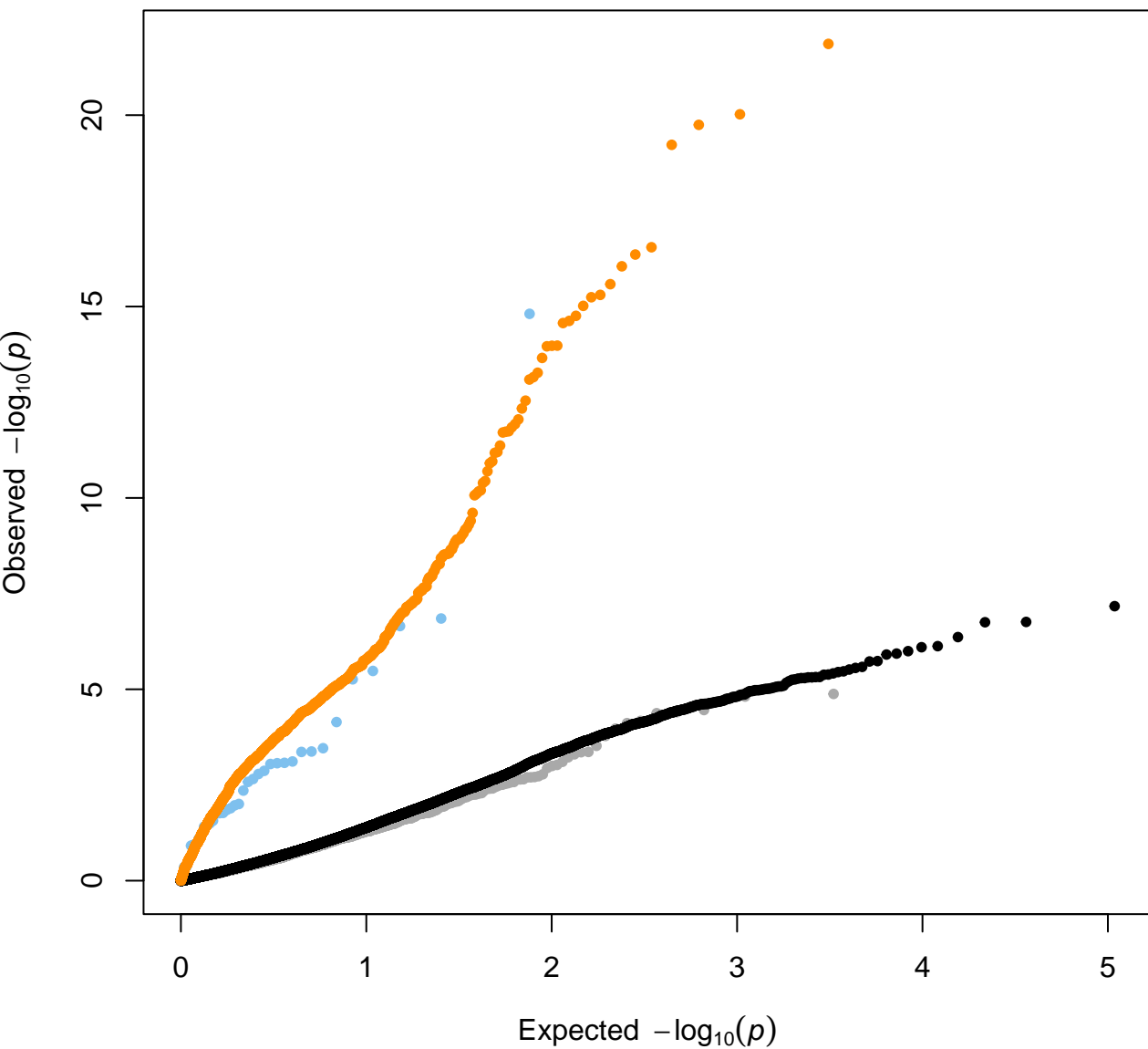

# IQ 16p

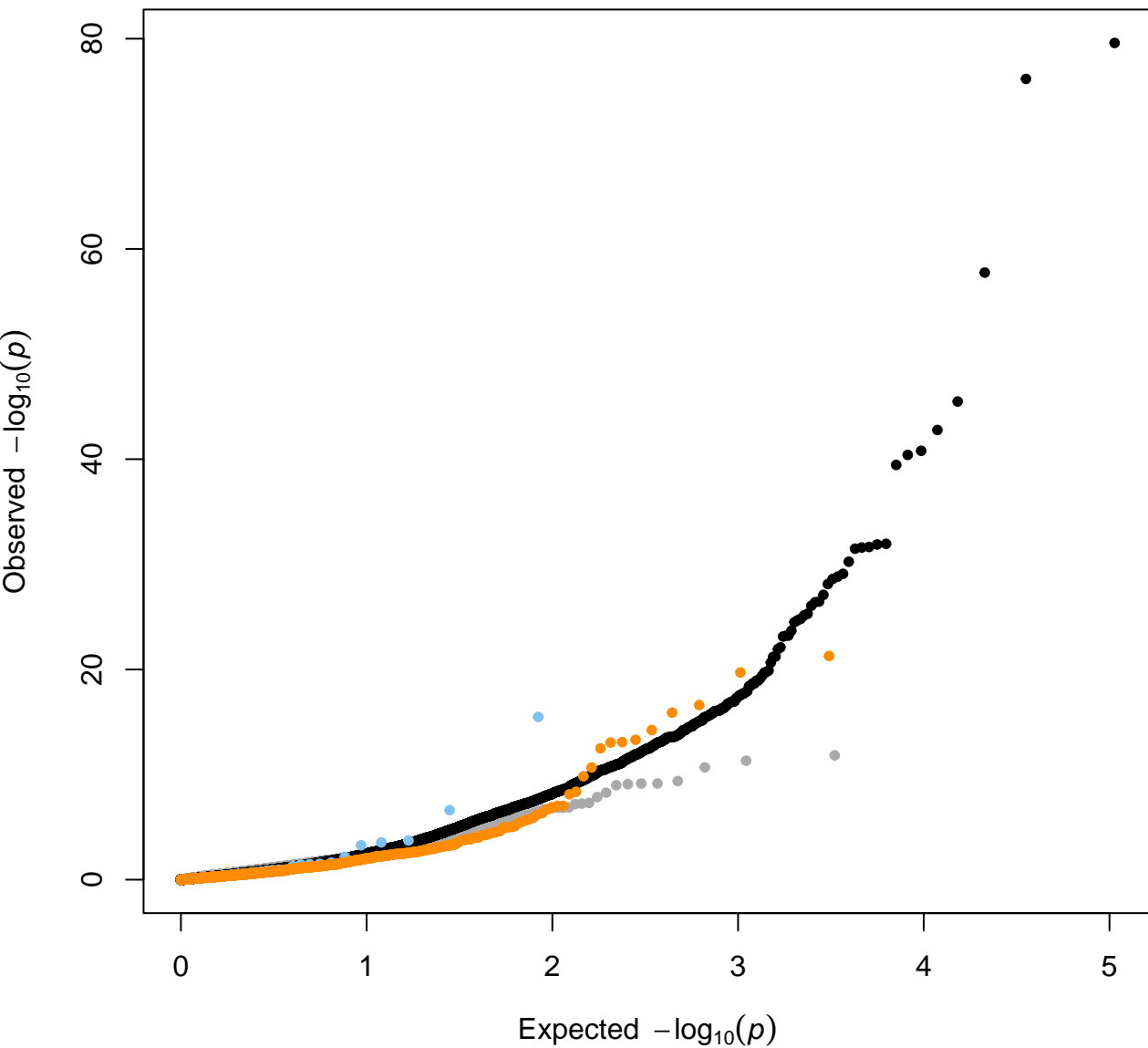

Supplement: S2 Fig — Q-Q plots comparing PrediXcan association signal from single 16p11.2 genes (blue), pairs of 16p11.2 genes (orange), single genes in control subsets (gray), and pairs of genes in control subsets (black). (PDF) [file pgen.1010780.s002.pdf]

# ASD 22q

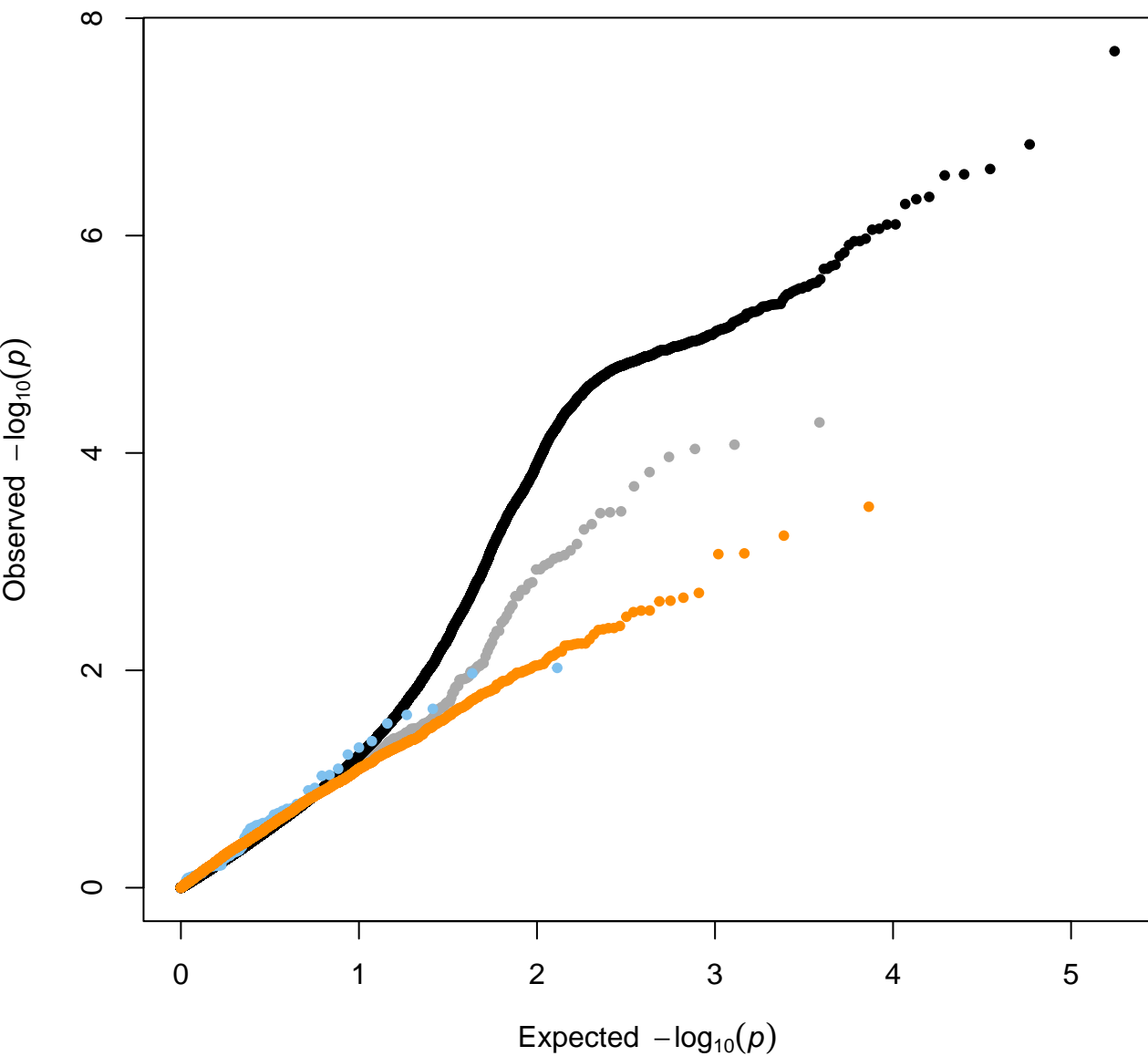

# BIP 22q

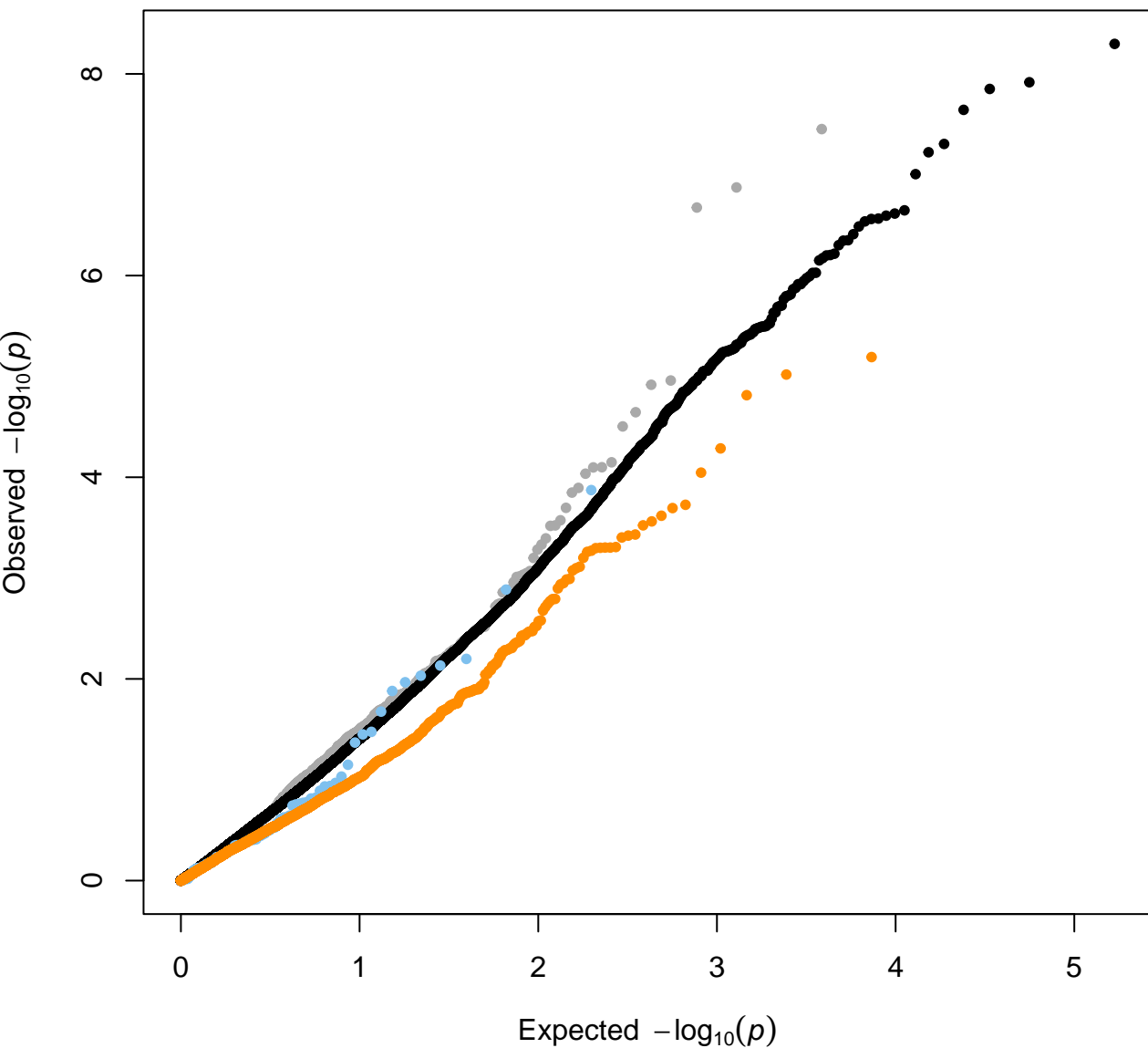

# SCZ 22q

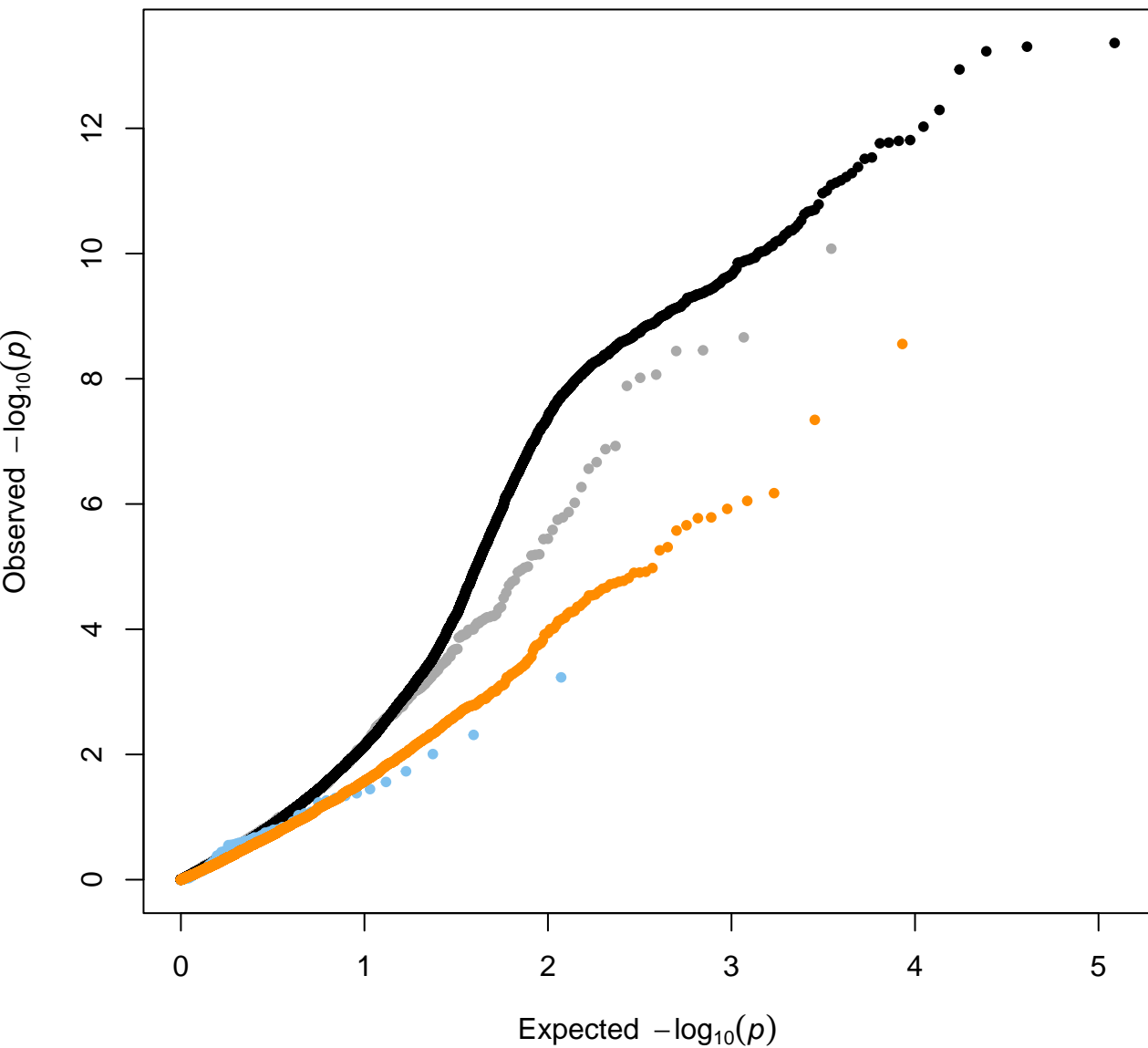

# BMI 22q

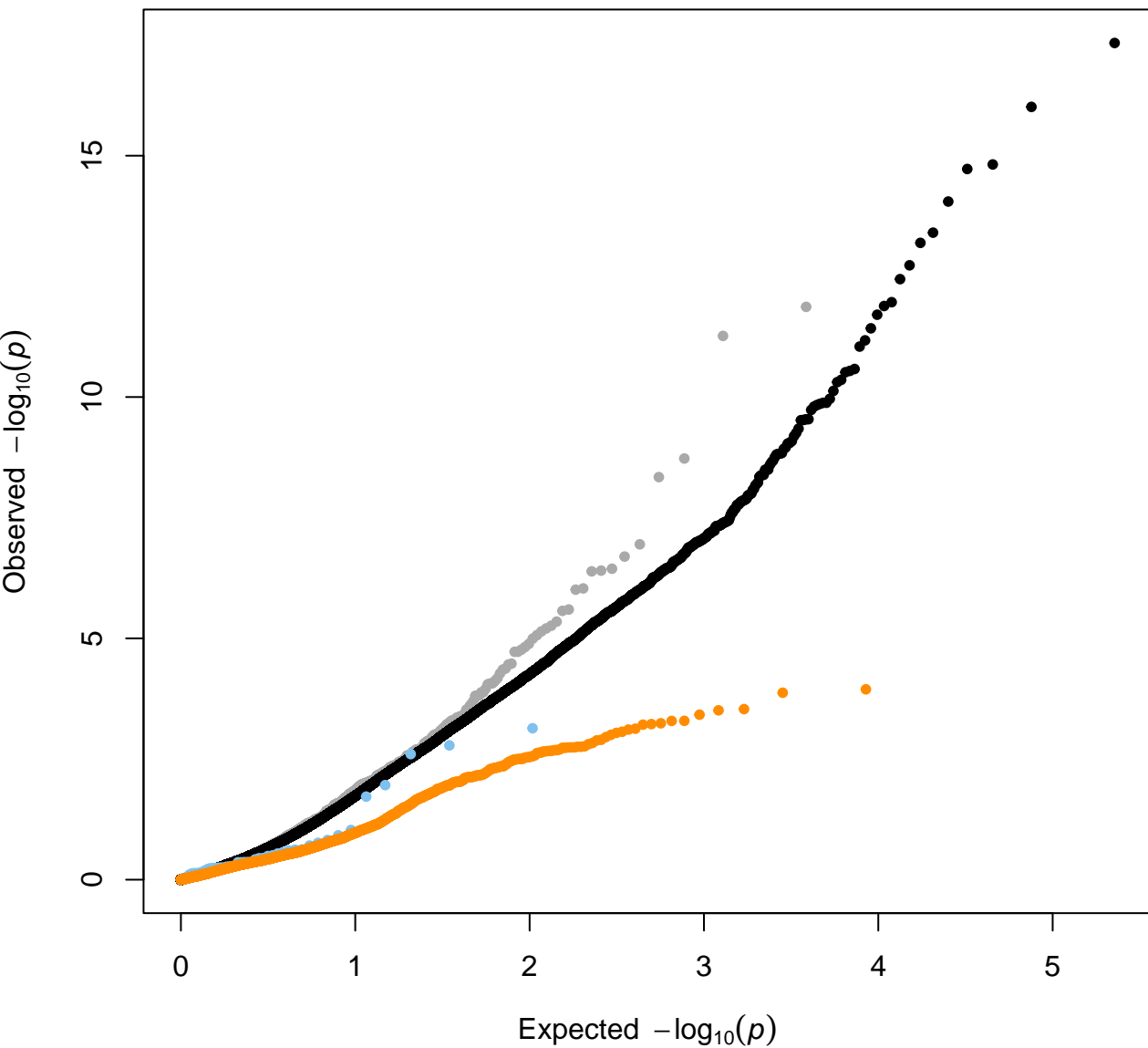

# IQ 22q

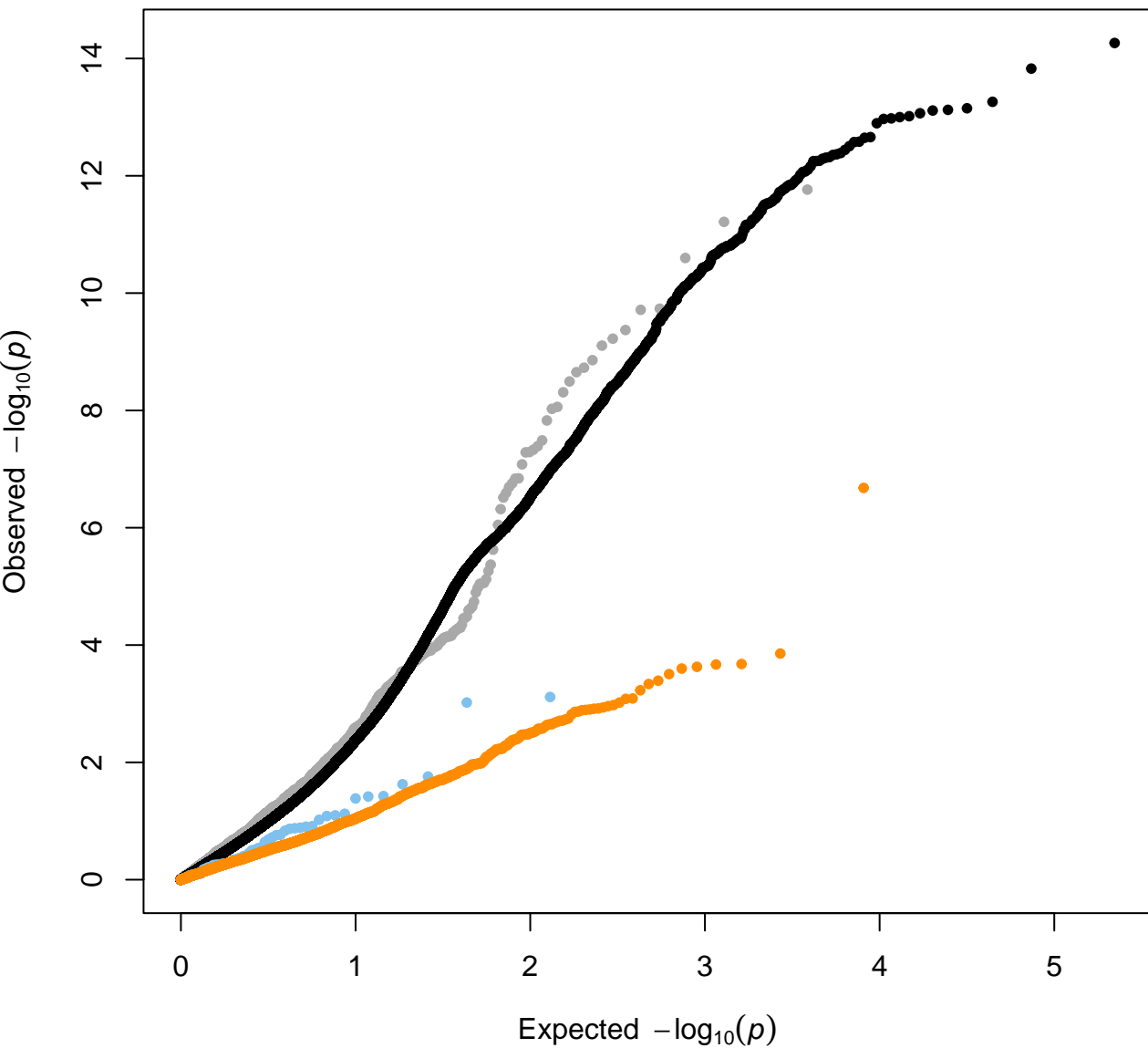

Supplement: S3 Fig — Q-Q plots comparing PrediXcan association signal from single 22q11.2 genes (blue), pairs of 22q11.2 genes (orange), single genes in control subsets (gray), and pairs of genes in control subsets (black). (PDF) [file pgen.1010780.s003.pdf]

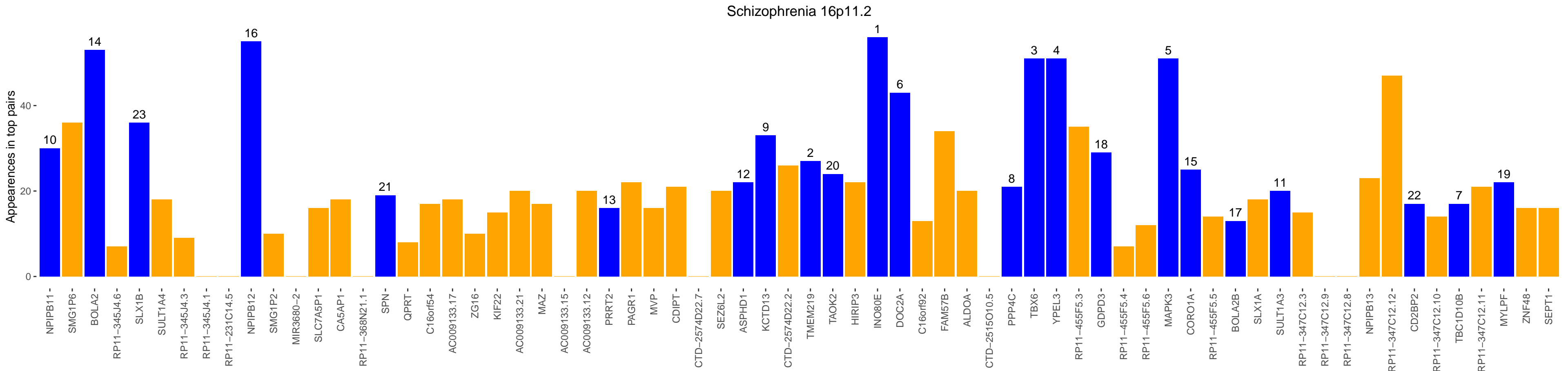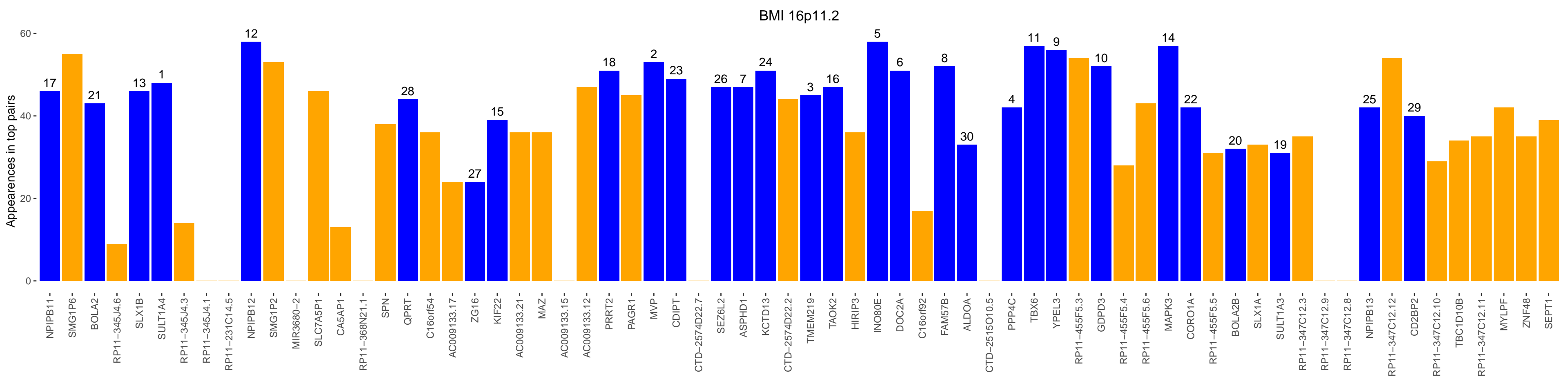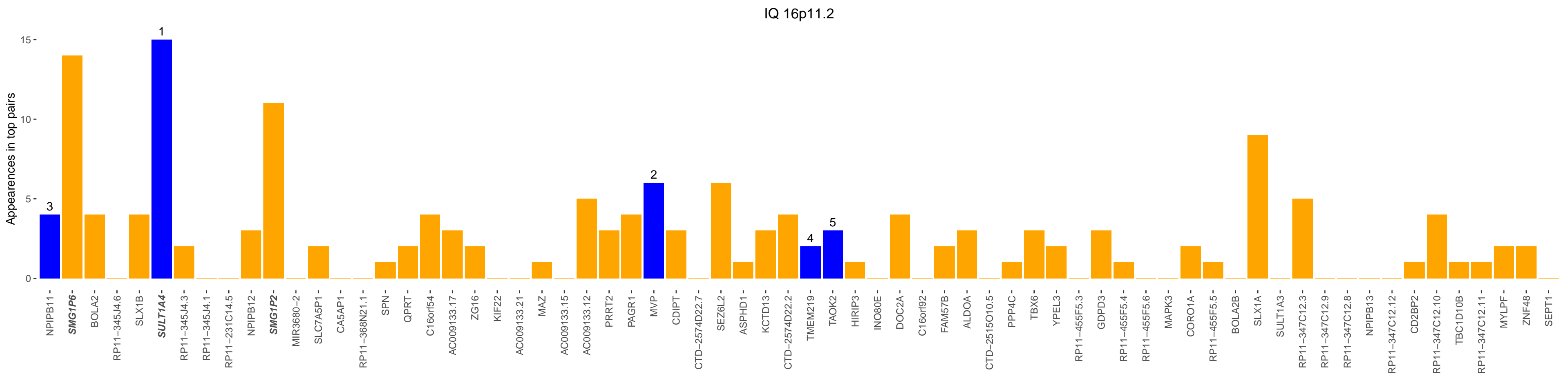

Supplement: S4 Fig — Y-axis: counts of the number of times each gene contributes to a significant pair (permutation P-value < median of 5th percentiles of control region p-values). Bars in blue represent genes significant (permutation P-value < median of 5th percentiles of control region p-values) in a single gene model for the same trait, with rank indicated above the bar. Bars in orange represent genes not significant in a single gene model. X-axis: genes in chromosomal order. Disproportionately represented genes (mean + 2.5 standard deviations) are bolded. (PDF) [file pgen.1010780.s004.pdf]

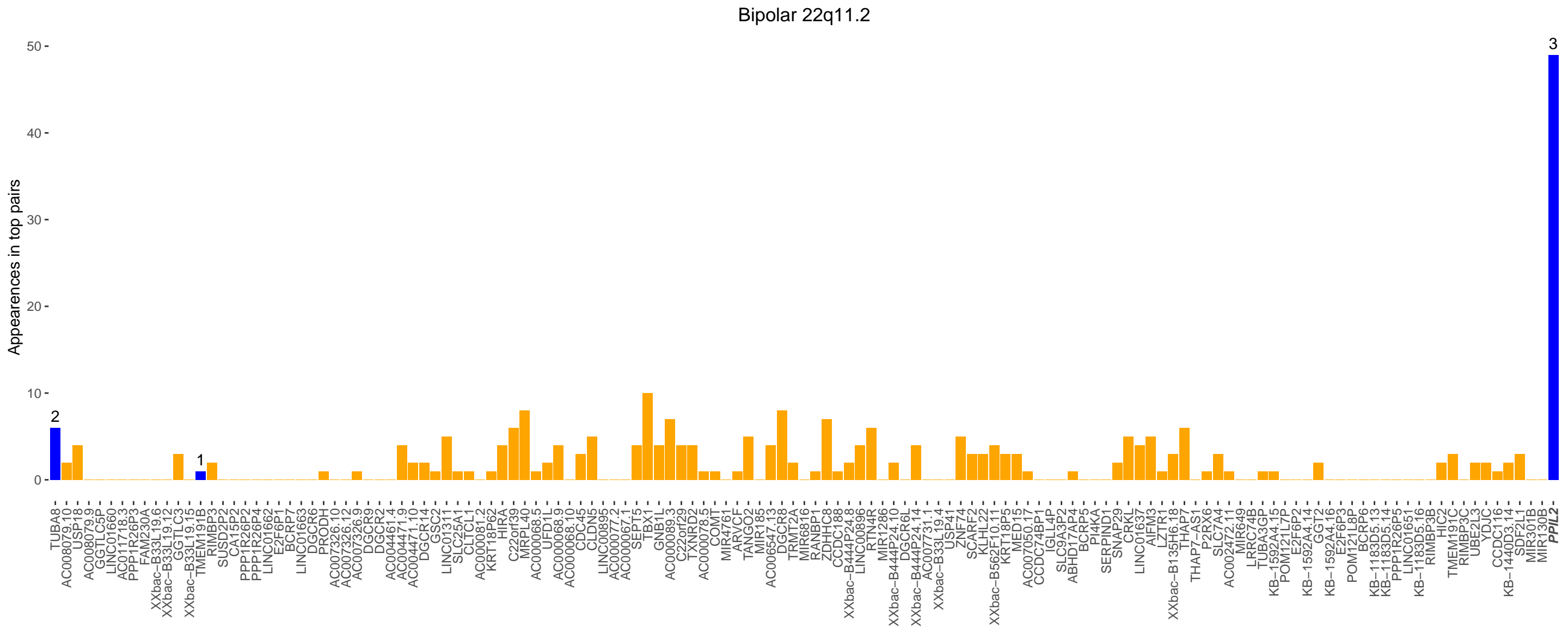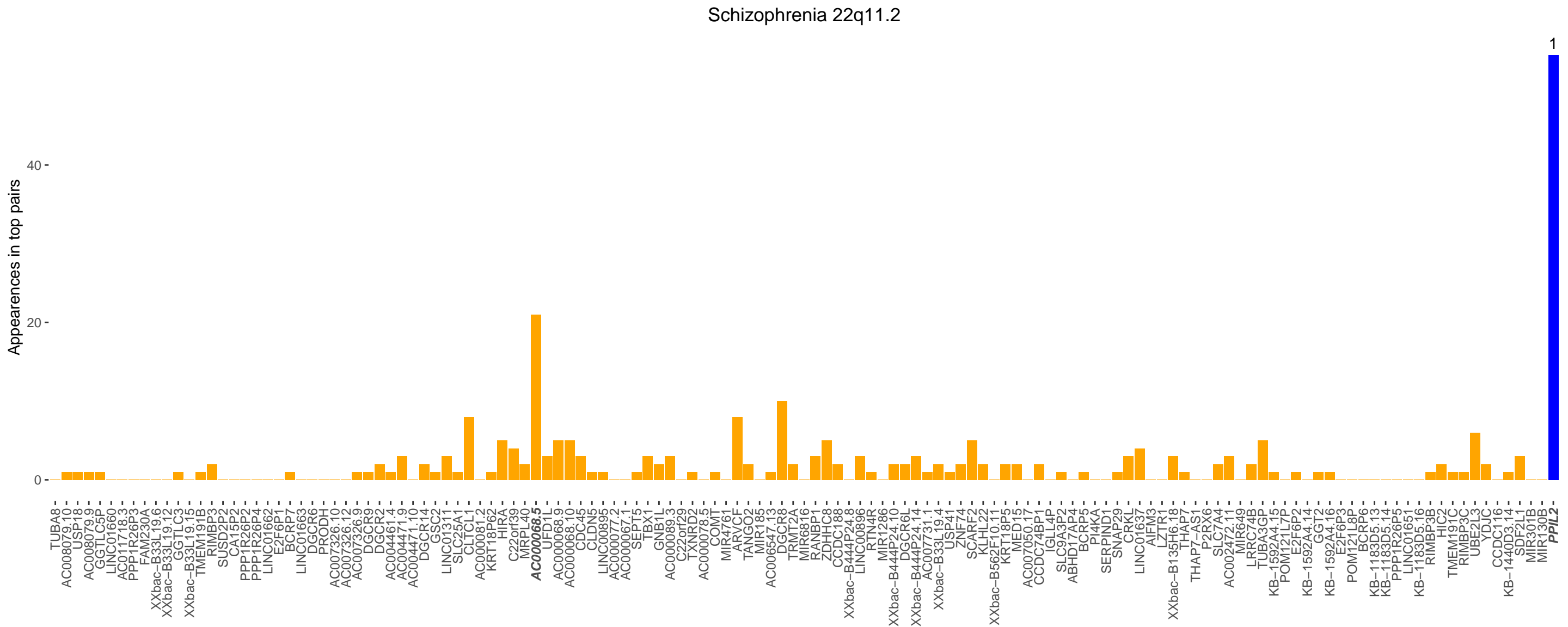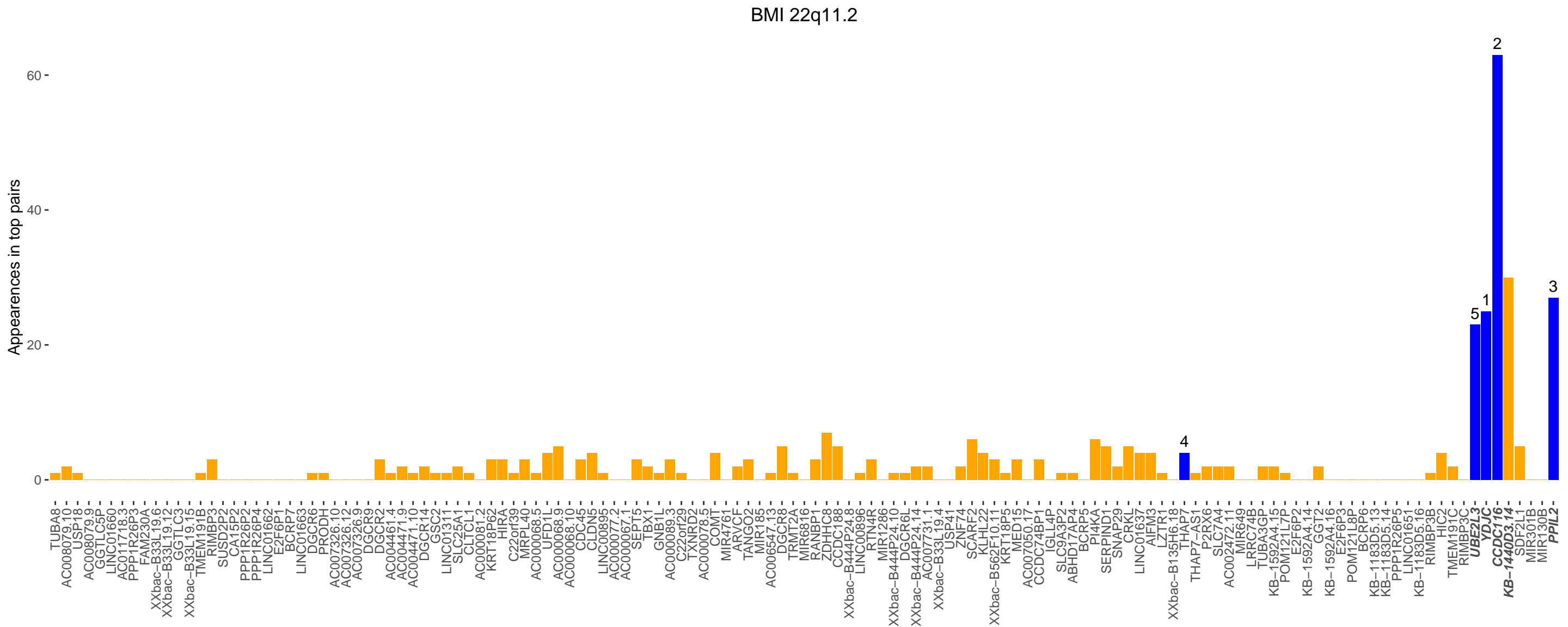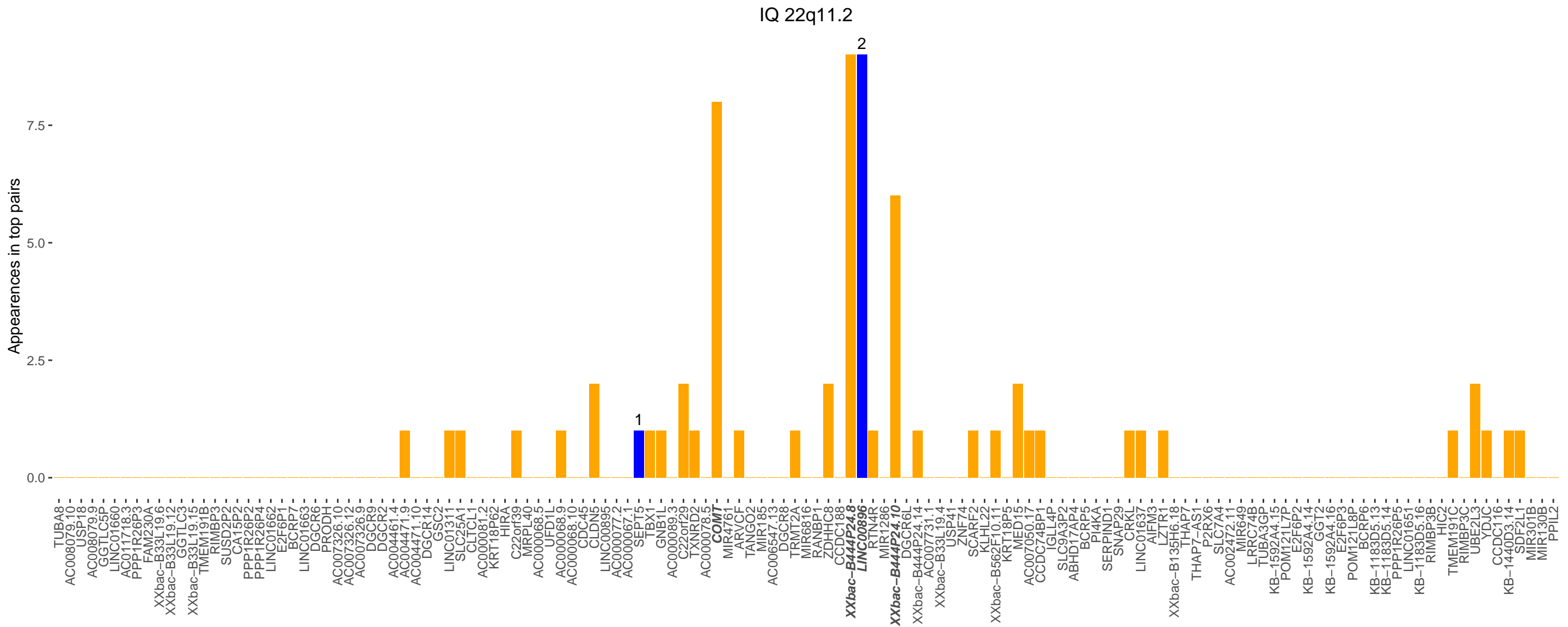

Supplement: S5 Fig — Y-axis: counts of the number of times each gene contributes to a significant pair (permutation P-value < median of 5th percentiles of control region p-values). Bars in blue represent genes significant (permutation P-value < median of 5th percentiles of control region p-values) in a single gene model for the same trait, with rank indicated above the bar. Bars in orange represent genes not significant in a single gene model. X-axis: genes in chromosomal order. Disproportionately represented genes (mean + 2.5 standard deviations) are bolded. (PDF) [file pgen.1010780.s005.pdf]
