# Supplementary material for: Combinations of genes at the 16p11.2 and 22q11.2 CNVs contribute to neurobehavioral traits
Source: PLoS Genet. 2023 Jun 2;19(6):e1010780. doi: 10.1371/journal.pgen.1010780 (PMC10266672; doi:10.1371/journal.pgen.1010780)
Supplement: S6 Fig — Region-wide scores across individuals were binned into deciles and the mean (dot) and standard error (bars) of case-control ratios for each decile are plotted. Best fit line across deciles is shown. Top: 16p11.2. Bottom: 22q11.2. Left to right: ASD, Schizophrenia, Bipolar Disorder. (PDF) [file pgen.1010780.s006.pdf]

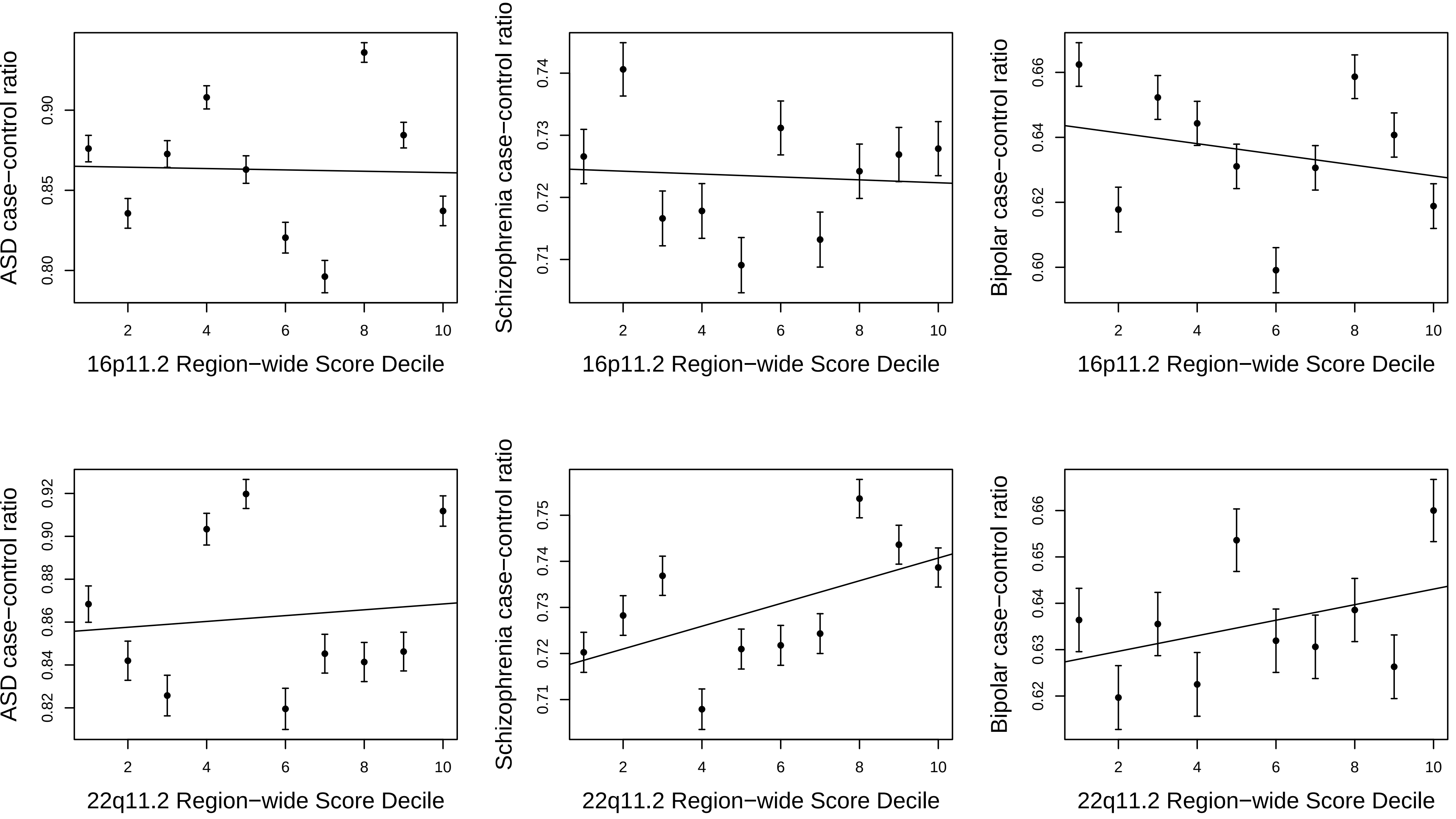
ASD case−control ratio
0.80
0.85
0.90
2
4
6
8
10
16p11.2 Region−wide Score Decile
Schizophrenia case−control ratio
0.71
0.72
0.73
0.74
16p11.2 Region−wide Score Decile
Bipolar case−control ratio
0.60
0.62
0.64
0.66
16p11.2 Region−wide Score Decile
ASD case−control ratio
0.82
0.84
0.86
0.88
0.90
0.92
22q11.2 Region−wide Score Decile
Schizophrenia case−control ratio
0.71
0.72
0.73
0.74
0.75
22q11.2 Region−wide Score Decile
Bipolar case−control ratio
0.62
0.63
0.64
0.65
0.66
22q11.2 Region−wide Score Decile
