# Supplementary material for: Combinations of genes at the 16p11.2 and 22q11.2 CNVs contribute to neurobehavioral traits
Source: PLoS Genet. 2023 Jun 2;19(6):e1010780. doi: 10.1371/journal.pgen.1010780 (PMC10266672; doi:10.1371/journal.pgen.1010780)
Supplement: Attachment — Submitted filename: reviewer_resp_22423.docx [file pgen.1010780.s016.docx]

Dear editors,

We enclose a revised version of PGENETICS-D-22-01128 “Combinations of genes at the 16p11.2 and 22q11.2 CNVs contribute to neurobehavioral traits.” In response to review, we have addressed further study limitations, improved our figure legends, and most notably, added Figure 1B, a flowchart representing our analysis framework. Our responses to specific reviewer comments follow.

Responses to first reviewer

I do have some questions about the premise and the approach, however. While the pairwise approach certainly makes more sense than investigation of single genes for these CNVs that elevate risk for highly polygenic traits, the expression-imputation methodology utilized here also rests on the critical assumption that genetic regulation is similar in cases and controls, which may not be the case. Further, the approach does not consider the vast regulatory landscape of both of these CNVs (e.g., https://www.medrxiv.org/content/10.1101/2022.03.23.22272826v1). 22q11.2 and 16p11.2 are genomic regions with the densest intrachromosomal contact; indeed 22q11.2 has the highest density of enhancer elements of any similarly sized genomic region. As such, broader disruptions of regulatory architecture may be explaining more of the variance in these neurobehavioral phenotypes than specific gene pairs in the loci.

*We agree wholeheartedly that our analysis does not capture all aspects of genetic regulation or CNV/case-specific regulation. However, our narrow restriction to cis-eQTLs present in controls has a number of advantages. In particular, when we observe significant results, we can be more confident in the causal relationship and specific genes involved. That said, we can’t draw many conclusions in the instances where we do not observe significance, so our approach may be prone to false negatives. This would be the case for chromosomal contact and enhancer mediated effects that contribute to CNV pathogenicity. It’s plausible that an enhancer or chromosomal contact locus within the CNV is* ***also*** *a cis-eQTL and our signal from the latter actually reflects pleiotropy with another functional effect in trans. In that scenario our general conclusions stand, i.e., pairs explain more variance than single ‘genes’, but the specific genes identified may be misleading. We have now addressed this possibility in our discussion. However, investigating these effects would require different methodology given the difficulty of trans-eQTL and trans-imputed expression studies and is thus beyond the scope of this study. We have now clarified our limitations with respect to false negatives.*

*In Discussion (p 16-17): “*By design, the expression imputation framework is based entirely on cis-regulation of expression and does not capture trans-regulatory effects. Thus an effect on trait that is due to non-cis architecture may be a false negative. A possible scenario is that a cis-eQTL has pleiotropy with a trans-regulatory region (such as chromosomal contact). If that is the case, within-region gene pairs that appear associated with phenotypes may be misleading with respect to specific genes, although our observations about the advantage of pairwise models for variance explained will still remain valid.”

The authors state in the Discussion that ‘perhaps [the] regulatory landscape across many regions of the genome also biases towards expression dysregulation of physically colocalized genes in the same direction’ ; which may be correct , but seems to go against their hypothesis; and then would also not explain why these genomic regions have such a uniquely strong contribution to neurobehavioral phenotypes.

*The reviewer notes some of the finer distinctions our use of control regions can pick up. Our primary hypothesis was that: multiple genes do a better job explaining the CNV association with a trait than a single gene in a CNV region. This was supported by our data for most CNV-traits comparing variance explained by pairs to by single genes (Table 2). A secondary hypothesis could be defined as: ‘pairwise’ biology is unique to CNVs (vs. reflecting the general genetic architecture of a trait). For this question, we can use control regions well-matched to CNV regions that should reflect the background ‘polygenicity’ for each trait. In this case, for 7 of 10 control-traits the pairwise model is also significantly preferred over the single-gene model, albeit for 5 of those 7, the preference for the CNV region is still more than for matched control regions, potentially due to specific pairs or above-average density of associated pairs (we added this note to the Discussion page 12). The observation of pairwise model preference in control regions leads to our interpretation quoted above about pairwise regulation generally being important to genetic architecture of association. The surprising aspect (to us) was that our model specifies both genes be dysregulated in the same direction, which makes sense for CNV regions but also tended to do better in control regions than more general interaction models. Given this general ‘same-direction’ local genetic architecture, it could help explain why CNVs are important in these traits, as they are a powerful same-direction hit to a number of nearby genes.*

On this point, I also am curious about their choice of these 5 phenotypes. There is no compelling association of bipolar disorder with these CNVs (certainly associations with other developmental neuropsychiatric disorders- ADHD, anxiety disorder- are stronger), and while there is one small study reporting on obesity in adults with 22q11.2 deletions this is a quite different level of evidence compared to the robust opposing anthropometric phenotypes observed for 16p11.2 reciprocal CNVs (e.g , Jacquemont et al, and other papers cited). Further, at least for BMI in 16p11.2 and for schizophrenia (possibly for both loci) there is evidence of opposing effects of the deletion vs duplication, but similar direction of effect for ASD and IQ. This doesn’t appear to be directly addressed in the manuscript.

*The 5 phenotypes we studied were chosen not only due to the magnitude of association with the CNVs but also for some practical reasons and for comparison with established data. For instance, BD has more data available than ADHD and anxiety disorder. In addition, there is a strong body of work on the genetic overlap across BD and SCZ, so we anticipated it being interesting to include both for comparison/specificity. Similarly, obesity was included for all CNVs so we would have equivalent comparisons available to interpret phenotype vs. CNV region specificity. Finally, this work is an extension of our prior single-gene and EMR biobank work with the same 5 traits (Vysotskiy et al).*

*We agree that reciprocal phenotypes at this region are important to consider. After working with eQTL/expression-imputation based approaches for several studies, we ultimately no longer consider direction of effect as meaningful/interpretable, despite our hope during study design that direction of expression regulation would be helpful for reciprocal phenotypes. The same observation has recently been made in Okamoto et al AJHG 2023. As this is a cross-tissue study, we find that eQTL directions change between different tissues, and in fact, the solution for combining across tissues (MultiXcan) tests an F-statistic which is directionless. We commented on this in the text on page 17: “*TWAS approaches are unable to capture direction of effect in a reliable way [36]. As a result, this experimental design also does not allow us to account for the interesting questions of directionality (i.e., in instances where a trait is present in only deletions or duplications) and reciprocal phenotypes.”

Given the lack of evidence for association of bipolar disorder with these CNVs, it is unclear how to interpret the distinct pattern for bipolar disorder compared to the other traits- ie, that at both loci, one gene (INO80E and PPIL2 respectively) stood out as a disproportionate contributor to pairs. Could the authors comment on this in the Discussion?

*We agree with the reviewer that evidence for association is weakest for bipolar disorder, although some data support a CNV-related risk increase in bipolar disorder, particularly at 16p11.2, referenced in Green et al, Mol Psych 2016, as well as 22q11.2, referenced in Patel et al Clin Psychopharma Neurosci 2022. Our other rationale for including bipolar disorder is because existing data suggest a high level of shared GWAS risk with schizophrenia, most recently described in the 2021 PGC Bipolar GWAS (Mullins et al). This has been added to Methods on page 20: “*Although bipolar disorder is not one of the strongest associated phenotypes with either CNV, it does get picked up in carrier screens, and we included it in the study due to its large sample size and high genetic overlap with schizophrenia.” *Setting aside individual genes, which are not the primary outcome of this study, bipolar disorder at 16p11.2 performed similarly to schizophrenia both in the proportion variance explained by pairs and by its advantage over control gene lists. At 22q11.2, bipolar disorder did not have a pairwise contribution exceeding a single gene contribution, so the top single gene(s) may be of further interest.*

Given that region-wide associations were only observed for the quantitative traits analyzed, is there any concern this could be a psychometric issue?

*While we are unable to disentangle any measurement issues from the results, the parsimonious explanation is the power advantage for quantitative over categorical traits in general, and the sample sizes in our data specifically. Our quantitative trait data sample sizes are one or more orders of magnitude larger than any of the binary traits. However, better measurement may reduce noise and further increase the power advantages.*

Also – was there any consistency or pattern to the types of genes (protein-coding, lincRNA, pseudogene, antisense, miRNA) showing strongest pairwise signal, across the 2 loci?

*Across most phenotypes, the vast majority of top hits were coding-coding pairs. (In Results page 8: “*Top pairs primarily consisted of two coding genes, consistent with the larger number of coding-coding pairs that had a high-quality prediction model.”) *This pattern is generally consistent with our ability to come up with quality prediction models, where coding-coding also was the most common model available by far. At 16p, pseudogene-coding was a distant second, while at 22q, lincRNA-coding was the second largest class. Out of curiosity, we tested whether the second-most common pair type was over-represented in top results with respect to coding-coding pairs, and we found that this was true for IQ at both 16p and 22q (binomial P < 0.05) and not for any other trait. We find this an interesting insight for follow-up studies of intelligence genetics. Discussion page 15: “*IQ is, in fact, the only trait for which coding-noncoding pairs are over-represented as significant hits with respect to coding-coding pairs, which may have implications for future genetic studies of IQ (Supplementary tables 1, 2, 6, 7)*.”*

Analyses were limited to those of ‘white’ ancestry – while perhaps this couldn’t be avoided, it certainly needs to be directly discussed as a limitation.

*We have included this as a comment in our limitations discussion on page 18: “*For consistency with the published Psychiatric Genomics Consortium data, we limited our analyses to white (European) ancestry. As a result, we are likely not capturing the entire spectrum of eQTL data and GWAS and are less confident in the transferability of our expression prediction models, but we expect that our findings about CNV-trait architecture will be true for any genetic-ancestry group.”

Responses to second reviewer

Using TWAS implemented in PrediXcan the authors attempt to understand the contribution of individual genes within two described microdeletion/duplication syndromes. The authors selected the genes encompassed by the syndromes for analysis (not specified), used summary statistics from the Psychiatric Genomics and GIANT consortia for various neuropsychiatric traits and conditions, and used predicted transcription levels from the updated GTEx v8 release. 38 control regions were selected for permutation-based analyses by number of genes, length, ratio of coding to non-coding genes, notably GC content was not accounted for.

*When selecting control genes/regions, it is difficult to match for every factor. The best metric, we believe, for determining that GC content or other unmatched properties are not leading to bias is by making sure that gene prediction quality (R^2^) is consistent with that of true sets. For 16p genes, the prediction median and mean are 0.091 and 0.12, respectively; for chosen controls they are 0.093 and 0.14. For 22q genes, the prediction median and mean are 0.1 and 0.13, respectively; for chosen controls they are 0.09 and 0.13. This consistency suggests that the controls are properly chosen and additional factors like GC content are unlikely to affect the results. We have now added this prediction quality matching to our methods section on page 22: “*For 16p11.2 genes, the prediction R^2^ median and mean are 0.091 and 0.12, respectively; for chosen controls they are 0.093 and 0.14. For 22q11.2 genes, the prediction median and mean are 0.1 and 0.13, respectively; for chosen controls they are 0.09 and 0.13. This consistency suggests that the control regions are well-matched for key properties relevant to predicted expression.

*As one final sanity check, we looked at the expression levels of the GTEx genes in the true list and controls to make sure the expression levels between CNV genes and controls were matching. For 16p genes, the median TPM expression was 1.36, compared to 1.04 in controls (about the 55^th^ percentile of control sets). For 22q genes, the median TPM expression was 0.21, compared to 0.23 in controls. Again, we find that the controls are consistent with the CNV genes.*


The abstract does not outline the methodological approaches with much clarity and does not highlight specific results of interest.

*We have tried to clarify and enhance the abstract.*

Some comment about the patient selection in the GWAS consortia is needed—it is likely that individuals with 22q11.2 deletion syndrome were included in these cohorts as the disorders are fairly common and underdiagnosed when congenital heart disease or thymic agenesis are not prominent phenotypes.

*Case selection is described in the PGC references cited in the manuscript as refs 25, 41, 42, 45, 46. It is true that patients with CNVs might be present in the sample for the reasons the reviewer describes. We know, however, based on measured prevalences across cohorts, that even within affected individuals, no more than 1-2% should be carrying a 16p or 22q CNV (for example, see Stefansson et al 2008, Weiss et al 2008, Grozeva et al 2012). Those individuals will have poor prediction of true expression level by SNP genotype, as the CNV presumably has a larger impact than the remaining allele(s), but the amount of noise added as a result will be negligible or bias towards false negative results and is unlikely to result in false positives or bias towards any particular model. In Methods, page 20: “*It is possible that 1-2% of the individuals in these cohorts are CNV carriers, resulting in poorer expression prediction, but the amount of noise will be negligible and biased towards false negatives rather than false positives.

The first sentence of the results should describe in a bit more detail the experimental approach used by the authors. Even after reading the methods I was still somewhat confused about the approach—was PrediXcan performed for each deletion/duplication gene along with the ‘control genes’ and the p-value derived from the permutation of these test-statistics? Was the significance threshold for the findings pre-specified? How were the pairwise signals calculated? An explanatory figure on the analytical approach and permutation procedure might be necessary for the general reader.

*There are two sets of genes our study analyzes: CNV genes (split into 16p and 22q) and corresponding matched controls. For each set, we applied single-gene prediction models (downloaded from Im Lab website) and pairwise prediction models (created by us) to generate single and pairwise predicted expression levels for individuals in GWAS of the relevant traits. These predicted expressions are then used for association analysis with five neurobehavioral traits. Association statistics for the control genes are used as a null distribution in order to find the significant associations of the true CNV genes. The majority of this article is the analysis of single and pairwise predicted expression levels and corresponding association statistics of CNV genes. The analysis flow has been better captured in Figure 1B.*

Figure 2:
Gene names are difficult to read and great in number. Would something like a regional Manhattan plot be more legible with genes annotated like the genome browser? The Y-axis “appearance in top pairs” – is not a familiar statistic to me. P-values with a clear explanation of their derivation from the permutation test might make more sense. A small note on style—I would encourage the authors to use something other than default ggplot colors.

*While we understand that there are a large number of genes by the definitions that we used, we think that it is important to show all of them in chromosomal order in order to reflect relative differences across genes. We have tried to generate this figure at high resolution so that it can be magnified in the electronic article version for legible gene names.*

*The count is not meant to be a statistic and there are no p-values or deviation from a null being tested. This is simply a number of times a gene appears in a pair passing the association cutoff. The one addition we made was to take the extreme high of these counts: genes for which the counts were 2.5 standard deviations from the mean and interpreted those as ‘highly represented in top pairs’.*

*We have rewritten the legend and changed the color scheme.*

Figure 3:
Further explanation is needed. The figure title notes an association but I do not see a statistical description of association or a p-value that would clearly indicate this interpretation. What is a region-wide score decile?

*The explanation for the region-wide score and the associated p-values are found in the text (page 25, methods; pages 11-12, results). Briefly, we scored each individual based on their median predicted gene expression across all CNV genes. Because of the large number of individuals in the UK Biobank dataset, we cannot reasonably display every individual, and so score deciles are used instead. To clarify for the figure, we expanded on the figure legend and added p-values there as well.*

Figure 4:
The title “Insights gained into CNV-trait pairs” should be more descriptive—what are the insights specifically?

*Perhaps “insights” was not the correct word to use here as this figure serves more as a results summary. We changed the figure title to “patterns” and edited the legend.*
