# Supplementary material for: Combinations of genes at the 16p11.2 and 22q11.2 CNVs contribute to neurobehavioral traits
Source: PLoS Genet. 2023 Jun 2;19(6):e1010780. doi: 10.1371/journal.pgen.1010780 (PMC10266672; doi:10.1371/journal.pgen.1010780)
Supplement: Attachment — Submitted filename: resp_reviewer_misha_42023.docx [file pgen.1010780.s017.docx]

To the Editor:

We enclose a revision of the manuscript *Combinations of genes at the 16p11.2 and 22q11.2 CNVs contribute to neurobehavioral traits* based on the latest round of review. In brief, we updated the text to de-emphasize individual gene pairs, added a supplementary table and figure to clarify the data represented in Figure 2, and added additional information to Figure 3.

Reviewer One

One remaining concern I have is the focus on the specific gene pairs in the discussion may be premature, given that -in the event of pleiotropy with trans-regulatory regions- the within-region gene pairs that appear associated with phenotypes ‘may be misleading with respect to specific genes’ (as the authors state in their response). We agree that the observations about the advantage of pairwise models for variance explained would still remain valid, but it would call into serious question any conclusions about the specific genes. Without further information about how likely this scenario is, I still think the conclusions about the specific gene pairs identified by this approach ought to be further tempered.

*We think that we are on the same page with the reviewer and have discussed these limitations in our previous review responses and the manuscript itself. We believe that our discussion of individual top pairs is of interest to CNV biologists; furthermore, independent support for genes/pairs we discuss from the literature helps the reader interpret our results in the context of prior work. We emphasize genes recurring among pairs or phenotypes, as we expect them to be more robust than individual pairs, and otherwise mention pairs consistent with literature or representative of a pattern. Still, it is important to be clear that candidate pairs could be false-positives, which we further clarified on page 14.*

“Given that the pairwise signal tended to be oligogenic and that expression imputation of adjacent genes has high correlation (as well as additional limitations of our genetic architecture analysis discussed below), it is difficult to interpret association at the level of specific gene pairs. Despite low confidence in any single result, we find it potentially informative to review literature support and potential future directions for our most statistically convincing gene (pair) associations, particularly those that recur across pairs or phenotypes or are representative of a pattern between individual and pairwise top results.”

*The reviewer’s suggested article, along with additional literature cited in the study, have been incorporated, as well (p.15). Further, we believe the additional supplementary figure suggested by Reviewer 2 demonstrating the interwoven predictor SNPs and LD structure for one example pair helps to clearly illustrate the challenge of correlation among genes that concerns the reviewer (Supplementary Figure 1).*

Reviewer Two

As I noted in the previous review, the representation of the data Figure 2 as essentially a large bar chart sorted in the order of the genomic locus is a missed opportunity. The data could either be more clearly conveyed in a table which would at least be more readable, or as a modified LocusZoom plot which would then show the relative length of each gene and give readers the opportunity to visualize how genes might be physically related to known cis-regulatory regions.

*We still believe that our figure is a helpful representation of the distribution of frequencies of occurrence in the top pairs, in order to interpret specific genes of potential interest and the relationship between individual gene signal and pairwise signal. We acknowledge that there are many datapoints in this figure due to the large gene number and our desire to visualize ‘overrepresentation’ among the full distribution. To clarify this, we changed the figure title (p. 29) to: “Three representative examples of the distribution and overrepresentation of individual genes involved in significant pairs”. The journal PLOS Genetics is an online-only journal where interested readers are able and encouraged to zoom in, when needed.*

*However, we also agree with the reviewer that there are other data of potential interest that are not very well-displayed by this figure. First, we tabulated the data from Figure 2 and added it to the supplement to make it easy to look up individual genes or sort the values, as suggested (Supplementary Table 8). We also expanded on the LocusZoom idea to illustrate the relationship between GWAS results, specific SNP predictors of each gene and their pair, and LD correlation (Supplementary Figure 1, results p. 8, methods p. 22, legend p. 33). As a key example, we took the top pair associated with schizophrenia at 16p11.2 (INO80E/ZNF48), and picked one potential schizophrenia-relevant tissue (frontal cortex). We selected the SNPs that were predictors for either INO80E, ZNF48, and/or the pair in the same tissue, and used LocusZoom to visualize the position of these SNPs relative to the genes and their association with schizophrenia; we paired this with a plot of the LD structure of this region. We found that predictor SNPs had a broad location distribution around the individual genes, co-localized with SNPs with schizophrenia association, and formed two distinct main LD-blocks. We hope this additional example figure adds a valuable dimension for the reader to understand the data underlying our results, and thank the reviewer for the suggestion.*

The information in Figure 3 also remains somewhat abstruse--this may be the most powerful set of information in the whole manuscript demonstrating (as I am understanding) that the predicted region-wide gene expression scores are related to some key continuous traits. I am honestly unsure how to convey the information more clearly--perhaps with a trendline relating the two variables for each region and an accompanying p-value?

*We added trendlines to the deciles plotted in Figure 3 and the respective supplementary figure (legends p. 31,35). Given that these figures summarize 500,000+ data points from the UK Biobank, we found the deciles to provide the clearest, most effective visualization for both the data distribution and trend. However, the p-values reported in the manuscript and in the legend come from the full 500,000+ individuals, so they aren’t derived from the summarized deciles or trendlines directly. For this reason, we opted to include the more accurate whole-cohort p-value (and explanation) in the legend to avoid a misunderstanding of what the p-value measures.*
